# Microscopic and Molecular Tracing of *Cryptosporidium* Oocysts: Identifying a Possible Reservoir of Infection in Red Grouse

**DOI:** 10.3390/pathogens6040057

**Published:** 2017-11-13

**Authors:** David Baines, Michaela Giles, Michael Richardson

**Affiliations:** 1 Game & Wildlife Conservation Trust, The Coach House, Eggleston Hall, Barnard Castle, Co. Durham DL12 0AG, UK; mrichardson@gwct.org.uk; 2 Animal and Plant Health Agency, Woodham Lane, Addlestone KT15 3NB, UK; Michaela.Giles@apha.gsi.gov.uk

**Keywords:** medicated grit, parasite, radio-tracking, respiratory cryptosporidiosis, *Trichostrongylus tenuis*

## Abstract

Infection by *Cryptosporidium baileyi* causes respiratory cryptosporidiosis in red grouse *Lagopus lagopus scotica.* First diagnosed in 2010, it has since been detected across half of moors managed for grouse shooting in northern England. We hypothesised that contaminated grouse faeces within communal trays visited by grouse containing grit coated with flubendazole, provided to control *Trichostrongylus tenuis* parasites of grouse, is a reservoir of infection. To establish the basis to this hypothesis, contents of 23 trays from a grouse moor were examined for *Cryptosporidium* oocysts. Contents were subjected to Immuno Magnetic Separation oocyst concentration techniques prior to examination by Immuno Fluorescence Antibody Test microscopy and molecular analysis on the 18S rRNA gene. Seven of 13 (54%) grit trays known to be used by infected grouse were positive for *Cryptosporidium* by IMS-IFAT, compared to two of 10 (20%) random background trays. Ten of the 13 (77%) trays used by infected birds amplified positive for *Cryptosporidium* by Polymerase Chain Reaction and three of the 10 (30%) random trays. All PCR amplified products sequenced matched with *C. baileyi*, with *C. parvum* also present in one tray. These data suggest that trays used to “worm” grouse may act as reservoirs of *Cryptosporidium* infection and their future design may need to be reconsidered to minimise contamination.

## 1. Introduction

Red grouse *Lagopus lagopus scotica* (hereafter grouse) are wild gamebirds of cultural and economic importance in the British uplands. They exhibit quasi-cyclical fluctuations in abundance over a four- to six-year period driven by the intestinal parasitic worm *Trichostrongylus tenuis* [1]. To reduce worm infestations in grouse, a benzimidazole-based anthelmintic, bound by a fat layer to grit particles (medicated grit) is provided as a substitute to naturally occurring grit, which is consumed daily by grouse to help digest heather *Calluna vulgaris*, their principal food. Medicated grit provided within each grouse territory is readily consumed by grouse, thus self-dosing against the worm. A revised grit formulation occurred in 2007. This involved a change of benzimidazole drug from fenbendazole to flubendazole, incorporation of a more temperature-resistant binding fat and a new mode of grit delivery-withdrawal using flip-lid trays, rather than being scattered on the ground. These changes have been associated with reduced worm infestations, apparent cessation of quasi-cyclical fluctuations in grouse abundance and a doubling of post-breeding grouse densities [2]. So successful has been the change in drug delivery that virtually all grouse managers now use this system.

Coincident with these improvements in parasite-grouse management, the first case of respiratory cryptosporidiosis was observed in grouse on a moor in the North Pennines, northern England in 2010, with the infecting cryptosporidia identified as *Cryptosporidium baileyi* [3]. By 2013, almost half of the 150 grouse moors in northern England were reporting signs of disease in grouse, characterised by swollen eyes and sinuses, with a mean prevalence of 4% [4]. These bulgy-eye symptoms, again confirmed as infection by *C. baileyi*, were associated with 51% lower survival and 43% lower breeding success in grouse and, should prevalence increase, have the capacity to markedly impact on income derived from grouse shooting [5]. Mechanisms under-pinning such rapid transmission between grouse across an entire region are unknown, but cryptosporidia oocysts transmission is usually faecal–oral, either through direct contact with infected hosts or through drinking water or eating food that is contaminated [6]. We hypothesised that recent changes to using communal grit trays, coupled with higher grouse densities, led to trays becoming contaminated by grouse faeces containing *C. baileyi* oocysts, thus contributing to the rapid manifestation of respiratory cryptosporidiosis across grouse moors in northern England. We considered whether grit trays used by known diseased grouse were more likely to contain *Cryptosporidium* oocysts than random trays from the same moor and thus supported our hypothesis. 

## 2. Results

Seven of 13 (54%) grit trays used by infected birds were positive for *Cryptosporidium* by Immuno Fluorescence Antibody Test-Immuno Magnetic Separation (IFAT-IMS), compared to two of 10 (20%) random background trays (Fisher’s Exact Test: *p* = 0.20) (Table 1). Ten of 13 (77%) of the trays used by infected birds tested positively by Polymerase Chain Reaction (PCR) and three of 10 (30%) random trays (Fisher’s Exact Test: *p* = 0.040). Sixty-three of 811 grouse (8%) caught showed signs of cryptosporidiosis. This prevalence did not differ significantly from the 20% frequency of oocyst contamination of background trays by IFAT-IMS (Fisher’s Exact Test: *p* = 0.18), but was lower than the 30% frequency by PCR (Fisher’s Exact Test: *p* = 0.040). Video footage at the 13 trays used by infected grouse showed no other bird or mammal visiting the trays, either incidentally or to consume grit. Only domestic sheep were recorded in the vicinity of trays. Of the three trays where grouse faeces from outside the tray were analysed separately from the contents of the tray, two trays and their associated nearby faeces were contaminated (IFAT-IMS and PCR). In the remaining tray, contamination was detected only by PCR, but not in the nearby faeces. The 800+ bp PCR amplified product in all positive samples was sequenced and identity confirmed using a BLAST search as 100% match to *C. baileyi* [Genbank Accession number AF093495], with one tray used by an infected grouse additionally showing the presence of *C. parvum*.

## 3. Discussion

Grit trays visited by diseased grouse were more likely to have contents contaminated by *C. baileyi* oocysts than random background trays, where it was unknown whether or not visiting grouse were diseased. Thus, trays may form an environmental contamination route and the potential for generating active infections within grouse themselves. Ingestion of 1 × 10^3^ *C. baileyi* oocysts per gram can result in intestinal cryptosporidiosis in poultry [7]. Whilst the minimum infectious dose to cause respiratory symptoms is undocumented, an orally inoculated dose rate of 1 × 10^5^ oocysts caused respiratory cryptosporidiosis in domestic chickens [8]. All positive grit tray samples, including faecal matter outside trays, had >1 × 10^3^ oocysts per gram, suggesting that grit trays and their immediate surroundings are a potential transmission source and a reservoir of infection. 

Despite demonstrating oocysts in tray contents, their viability was unknown. Persistence of *C. parvum* oocysts from livestock depends on temperature and soil types [9], with retained infectivity down to −22 °C [10]. Such extreme low temperatures were not recorded during our study and high rainfall, together with poor tray drainage, suggest desiccation is also unlikely to contribute to reduced viability. We thus assume that the oocysts observed were viable.

Oocysts are the only *Cryptosporidium* life-cycle stage involved in disease transmission and their minimization is key to controlling infection. They are however resistant to environmental stress and to disinfectants commonly used in poultry facilities, where control relies on rigorous nutritional and sanitary management that reduces exposure to oocysts [11]. To reduce infection in grouse, whilst still actively controlling *T. tenuis* worms, it may be necessary to redesign receptacles that hold medicated grit. Assuming that a source of contamination is through oocysts contained within grouse faeces deposited within the tray, any revised design should aim to reduce this risk by minimising defaecation within the container. Based on the same assumption, such dispensers should be moved regularly to avoid faecal contamination in their immediate vicinity. Trays may not be the only source of contamination and further sampling, including that of multiple small natural moorland pools, which grouse visit to drink, especially in dry periods, needs to be implemented. Grit trays, unlike moorland pools within internationally protected habitats, due to their high degree of usage by grouse, may however form a more logical start point to direct sanitary management aimed to reduce infection. 

## 4. Materials and Methods

As part of a study into impacts of respiratory cryptosporidiosis on grouse productivity and survival [5], 811 grouse were caught at night on a moor in the North Pennines, northern England and inspected for macroscopic signs of respiratory cryptosporidiosis [3]. Of these, 120 were equipped with necklace mounted radio-transmitters. Repeat locations of a sample of diseased radio-tagged grouse were used to define the spatial extent of breeding territories. Gritting trays, typically spaced at 100-m intervals, within each sample territory were identified and a trail camera positioned beside the tray to record whether or not it was visited by the diseased radio-tagged territory occupier. In this way, gritting trays visited by 13 diseased individuals were identified, three in spring 2014 and 10 in spring 2015. 

Those tray contents, including medicated grit and grouse faeces, were collected. In 2014, grit and grouse faeces collected from within the tray itself and faeces from within 30 cm of the tray were stored and analysed separately. In 2015, tray contents (grit and faeces) only were collected; 10 visited by diseased individuals and a further 10 random trays on the same moor as background samples, where the presence of faeces indicated that grouse visited the trays, but where the disease status of those birds was unknown. 

One gram of grit from each tray was washed in 10 ml of sterile water for 15 min. The grit was allowed to settle and the resultant supernatant was collected. This was subjected to the IMS concentration technique, using a Dynabead anti-Cryptosporidium IMS kit in duplicate [12]. One of the duplicates was sent for IFAT microscopy analysis. The antibody used binds to all species of cryptosporidium oocyst [13]. The remaining duplicate IMS extracted samples were DNA-extracted using the Qiagen mini-stool kit following additional freeze/thaw treatment of any IMS extracted oocysts. The extracted DNA (where present) was amplified using a nested 18S rRNA PCR visualised on a 1.8% electrophoresis gel using the revised reverse primer [14], thus allowing an increase in sensitivity of oocyst detection of several orders of magnitude over the conventional corpodiagnostic IFAT method [12], which has been demonstrated to be as few as one oocyst [15]. Positive amplified product was further analysed by ABI sequencing and all positive results compared to the Genbank NCBI database to determine the species of *Cryptosporidium* present in the original sample. 

## Figures and Tables

**Table 1:** Results from Immuno Fluorescence Antibody Test-Immnuo Magnetic Separation tests of contents of 10 grit trays used by individual red grouse infected with respiratory cryptosporidiosis (tray code denotes radio-transmitter frequency of visiting grouse) and 10 random background grit trays sampled in 2015, together with the outcome of Polymerase Chain Reaction amplification and the *Cryptosporidium* species identified. Data from three grit trays in 2014 used by infected grouse plus faeces within 30 cm of the tray are also included. (+ = positive, − = negative).

Year	Tray Code	IFAT-IMS	Oocysts/Gram	PCR	Sp. Present
2015	182	+	1 × 10^3^–1 × 10^4^	+	*C. baileyi*
	812	+	1 × 10^3^–1 × 10^4^	+	*C. baileyi*
	139	+	1 × 10^3^–1 × 10^4^	+	*C. baileyi*
	713	+	1 × 10^3^–1 × 10^4^	+	*C. baileyi*
	290	+	1 × 10^3^–1 × 10^4^	+	*C. baileyi*
	77	−	<1 × 10^3^	+	*C. baileyi*
	918	−	<1 × 10^3^	+	*C. baileyi*, *C. parvum*
	971	−	<1 × 10^3^	−	None
	512	−	<1 × 10^3^	−	None
	503	−	<1 × 10^3^	−	None
	Background	+	1 × 10^3^–1 × 10^4^	+	weak *C. baileyi*
	Background	+	1 × 10^3^–1 × 10^4^	+	*C. baileyi*
	Background	−	<1 × 10^3^	+	*C. baileyi*
	Background	−	<1 × 10^3^	−	None
	Background	−	<1 × 10^3^	−	None
	Background	−	<1 × 10^3^	−	None
	Background	−	<1 × 10^3^	−	None
	Background	−	<1 × 10^3^	−	None
	Background	−	<1 × 10^3^	−	None
	Background	−	<1 × 10^3^	−	None
2014	1	−	<1 × 10^3^	+	weak *C. baileyi*
	1 (faeces)	−	<1 × 10^3^	−	None
	2	+	1 × 10^3^–1 × 10^4^	+	*C. baileyi*
	2 (faeces)	+	1 × 10^3^–1 × 10^4^	+	*C. baileyi*
	3	+	1 × 10^3^–1 × 10^4^	+	*C. baileyi*
	3 (faeces)	+	1 × 10^4^–1 × 10^5^	+	*C. baileyi*
